# Diagnosing limb paresis and paralysis in sheep

**DOI:** 10.1136/inp.h5547

**Published:** 2015-11

**Authors:** James Patrick Crilly, Nina Rzechorzek, Philip Scott

## Abstract

Paresis and paralysis are uncommon problems in sheep but are likely to prompt farmers to seek veterinary advice. A thorough and logical approach can aid in determining the cause of the problem and highlighting the benefit of veterinary involvement. While this may not necessarily alter the prognosis for an individual animal, it can help in formulating preventive measures and avoid the costs – both in economic and in welfare terms – of misdirected treatment. Distinguishing between central and peripheral lesions is most important, as the relative prognoses are markedly different, and this can often be achieved with minimal equipment. This article describes an approach to performing a neurological examination of the ovine trunk and limbs, the ancillary tests available and the common and important causes of paresis and paralysis in sheep.

PARESIS may be defined as ‘a deficiency in the generation of the gait or in the ability to support weight’ and implies that a degree of voluntary movement is still present. Paralysis (plegia) is the complete loss of voluntary movement. Muscle weakness is associated with both conditions but may also be due to other causes.

## History taking

With reference to the chief complaint, it is important to ask the farmer about onset, duration, evolution (static, progressive, waxing and waning, episodic) and lateralisation of clinical signs (unilateral, bilateral, more marked on the right or left). Ascertaining which animals and how many are affected is also important, as are any relevant husbandry factors. Examples include:

▪ What are the sheep currently being fed? Has the diet changed recently?

▪ Are there known/suspected mineral/trace element deficiencies on the farm?

▪ Has the affected animal/group/flock received any treatments recently?

▪ Is the flock open or closed?

▪ What is the origin of the affected animals?

## Neurological examination

The core objectives of a neurological examination, together with the history, are to determine:

▪ Whether the condition is neurological;

▪ What part of the nervous system is affected;

▪ A list of differential diagnoses for the clinical signs observed;

▪ An appreciation of the severity of the disease (in order to give a prognosis).

A neurological examination should always be accom-panied by a full clinical examination to avoid missing non-neurological causes of the presenting complaint (eg, orthopaedic), as well as those that manifest in neurological signs secondary to generalised metabolic, infectious, toxic or nutritional disturbances (Box 1).

Box 1: **Common causes of recumbency in sheep**

It is important to determine whether a recumbent sheep is down due to a primary nervous system lesion or is affected by a different problem that may be unrelated to the nervous system or that may affect it indirectly. Examples of commonly encountered causes of recumbency include:

**Primary neurological disorders**

▪ Listeriosis (infection of the trigeminal nerve and associated brainstem nuclei by *Listeria monocytogenes*);

▪ Spinal cord trauma.

**Systemic disturbances that affect the nervous system (secondary neurological disorders)**

▪ Hypocalcaemia (see Video 1);

▪ Hypomagnesaemia;

▪ Polioencephalomalacia (cerebrocortical necrosis), eg, thiamine deficiency.

**Non-neurological problems**

▪ Multiple limb lameness;

▪ Severe debility and emaciation*;

▪ Septicaemic and toxaemic states (eg, acute mastitis, metritis, clostridial disease)*;

▪ Severe pneumonia;

▪ Acute fasciolosis.

* These conditions may present with secondary neurological disorders

**Figure:**
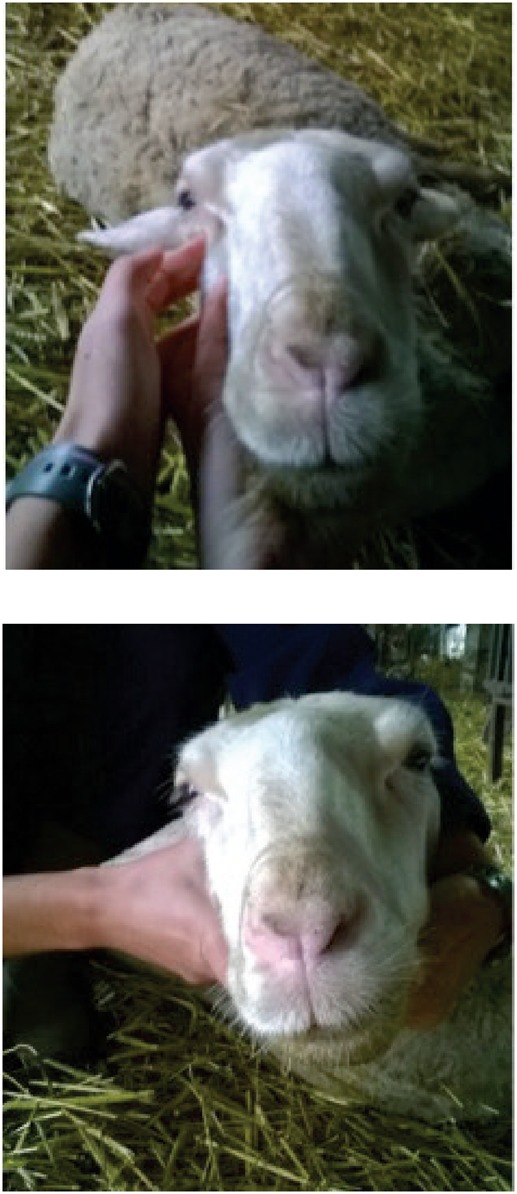
This ewe is recumbent due to listeriosis but the changes to facial symmetry are subtle (minor changes in the shape of the ocular and nasal aperture and set of the lip and ear) and the animal may be misdiagnosed if the cranial nerve function were not assessed

### Initial assessment

An initial assessment should be made from a distance. The demeanour, stance and posture of the animal should be observed. If it is recumbent, is there opisthotonos (marked dorsal extension of the head and neck)? Do the limbs appear flaccid or rigidly extended? Does it display the Schiff–Sherrington phenomenon (Table 3)? If it is able, the animal should be encouraged to stand, walk and turn, and the following assessed:

▪ What stance does the animal adopt? Is it wide based or narrow based?

▪ What is the posture? Is there kyphosis (dorsal curvature of the spine), lordosis (ventral curvature of the spine), scoliosis (lateral deviation of the spine), torticollis (twisting of the neck) or an abnormal head carriage? Are the elbows, hocks or fetlocks dropped?

▪ Is the face symmetrical? If not, which areas are affected and how do they differ?

▪ Is the gait normal?

- Is there any ataxia (poor coordination when moving) and which limbs are affected?
- Is the protraction–retraction of each limb normal, hypometric or hypermetric?
- Is the gait stiff or stilted?
- Is there any paresis or plegia? (Note, toe dragging indicates paresis; this is sometimes more easily heard than seen but hoof scuffing may give clues);
- Does the problem affect one limb, a pair of limbs or all four limbs?

▪ If the animal circles, are the circles tight or wide and in which direction?

Animals should be observed for involuntary movements such as tremor (involuntary oscillating contraction of antagonistic muscle groups) or myoclonus (sudden contraction followed by immediate relaxation of a specific muscle group).

The assessment should then be followed by a general clinical examination and a specific neurological examination. Particular attention should focus on eliminating other causes if a neurological problem is suspected. For example, muscle wasting of a limb may be the result of prolonged disuse due to an orthopaedic cause of lameness, and cardiovascular or respiratory embarrassment may be responsible for episodes of collapse. Important differential diagnoses for recumbency (other than those covered in this article) are given in Box 1.

### Localising the lesion

A neuroanatomical diagnosis is required before listing differential diagnoses of any neurological problem. Certain problems/pathologies are more common at particular sites than others. A systematic approach using only very basic tools can yield accurate information about a lesion's location and distribution (whether it is focal, multifocal or diffuse, and symmetrical or asymmetrical). The priority is to identify a single site that explains all of the neurological signs observed, since multifocal lesions are less common.

The stages of the close-up neurological examination can be performed in any order but should be methodical and consistent to avoid missing important information. The authors find it easiest to start at the head with assessment of mentation and behaviour, abnormal head carriage, head tremor, head turn or head tilt, facial symmetry and more specific testing of the cranial nerves (Table 1). When assessing the indicators of cranial nerve function, it must be remembered that non-neurological causes of abnormalities are also possible (eg, facial symmetry disrupted by swelling and tongue position, and movement disrupted by injury). The gag reflex is difficult to assess in sheep due to the narrow oral aperture and long buccal cavity. Facial and trigeminal nerve dysfunction is often accompanied by food packing into the cheeks, and the corneal reflex is a reliable test of cranial nerve VI function only if the eyelids are held open to observe the retraction of the globe.

**Table 1:** Tests of cranial nerve function

Test/observation	Cranial nerve involved
Facial symmetry	VII
Ear position	VII
Ocular aperture	VII
Eye position	III, IV, VI
Auditory response (eg, to a hand clap)	VIII
Menace response	II, VII
Palpebral reflex	V, VII
Corneal reflex	V, VII, VI
Pupillary light reflex	II, III
Facial sensation	V
Jaw tone	V
Tongue tone and position	XII
Vestibulo-ocular reflex	VIII, III, VI, IV

The range of motion of the neck or a pain response elicited on manipulation or palpation of the neck, back, muscles or joints may identify a region for further investigation. Subsequently, if the animal is standing, tests of proprioception, such as a modified ‘paper-slide test’, should be performed (Box 2).

Box 2: **Testing postural reactions and spinal reflexes**

**Proprioceptive positioning**

It is more difficult to test postural reactions in sheep than in small animals or horses because they are less tolerant of their limbs being handled. The authors find the easiest method is to place a foot on a robust piece of plastic (eg, an old feed sack) and, ensuring the animal is weight bearing on that limb, drawing the plastic laterally to assess how rapidly the animal detects the displacement of the limb and how accurately it returns the limb to the accustomed position. Hopping tests, wheelbarrow tests, hemiwalking, and visual and tactile placing tests as performed in small animals can be performed with lambs and small adult sheep

**Cutaneous trunci (panniculus) reflex**

This reflex has a segmental input and a unitary output at the level of C8-T1 running to the cutaneous trunci muscle of the flank. In response to a light stimulus (eg, a fly landing on the animal's side), the cutaneous trunci contracts several times. It is best stimulated by gently pinching the skin in the region of the sublumbar fossa and the corresponding area of the thoracic flank (it can frequently be elicited by touching the tip of a ballpoint pen on the skin). Sequential stimulation of the flank from the caudal to cranial aspect can help locate a spinal lesion, ie, the cutaneous trunci reflex cannot be stimulated below the lesion but is intact above it. Due to the overlap of dermatomes, precise localisation (ie, to a single nerve root segment) is often not possible

**Perineal reflex**

To assess the perineal reflex, the skin of the perineal region is pinched firmly. A normal response entails contraction of the anal sphincter and ventroflexion of the tail, and should occur irrespective of which side of the perineum is pinched. A reduced response indicates damage to the sacral spinal cord segments or damage to the pudendal and caudal rectal nerves (Constable 2004)

**Myotatic reflexes**

Tests for myotatic reflexes involve an afferent (sensory) arm and an efferent (motor) arm that often involve the same nerve trunk; both arms communicate in the same spinal segment. Decreased reflexes are due to muscular disorders, peripheral neuropathies or damage to the spinal segment where the nerve(s) involved originate. An exaggerated reflex is most frequently due to a lack of descending inhibition resulting from a spinal lesion cranial to the spinal segment of nerve origin, but it may also be exaggerated if the antagonistic muscle tone is reduced. Clonus indicates a lack of descending inhibition. The most commonly used tests involve the following:

▪ Patellar reflex. This reflex tests the integrity of spinal cord segments L4-L6 and the femoral nerve. The stifle is flexed to tense the patellar tendon and the crus rested on an open palm (to avoid restricting its movement). The patellar tendon is struck with a narrow, hard object (eg, the handle of a pair of scissors). The intact reflex produces a rapid contraction of the quadriceps and extension of the stifle. When performing this test, it may help to displace the skin of the limb laterally to ensure that woolless skin overlies the tendon. Striking the muscle body or the bones will produce misleading results;

▪ Extensor carpi radialis (ECR) reflex. This reflex tests the integrity of spinal cord segments C7-T2. The carpus is flexed and the metacarpus rested on the open palm. The front of the carpus is palpated and the tendon of the ECR located. This is again lightly and sharply struck, and contraction of the ECR results in an extension of the carpus;

▪ Tendons of gastrocnemius (testing L7-S1 and the tibial nerve), biceps brachii (testing C6-C8), triceps brachii (testing C6-C8) and the muscle belly of the cranial tibial muscle (testing L6-S1 and the peroneal nerve) may be struck, but the authors find the response difficult to reliably produce in neurologically normal animals.

**Withdrawal reflexes**

A painful stimulus to the distal limb (eg, pinching between the toes with artery forceps) will produce a withdrawal of the limb and should elicit flexion at all joints of that limb. This involves multiple motor units and therefore tests the integrity of a single sensory input but multiple motor outputs. A neurologically normal animal should be conscious of the stimulus as well but as sheep are stoic animals this may be hard to judge

Once the ability to withdraw the limb has been established, stimulation of different parts of the limb (eg, using a pen or forceps) can be used to assess the integrity of the sensory innervation to different parts of the limb. The crossed extensor reflex (extension of the contralateral limb when the withdrawal reflex is stimulated) is normal in neonates but indicates an upper motor neuron lesion if seen in an adult

The animal can then be laid on its side and the spinal reflexes of the uppermost limbs tested, after which the animal should be turned over and the tests repeated on the contralateral limbs. Other spinal reflexes can be tested while the animal is standing or recumbent and are described in Box 2.

Urinary function is an important indicator of caudal spinal cord disorders but is hard to assess in sheep. Has the farmer seen the animal urinate recently? Some animals will void the bladder if the nostrils are covered but this test should be performed at the end of the examination as it will distress the animal. Transabdominal ultrasonography (inguinal window) allows bladder size to be measured (Scott 2012).

**Fig 1:**
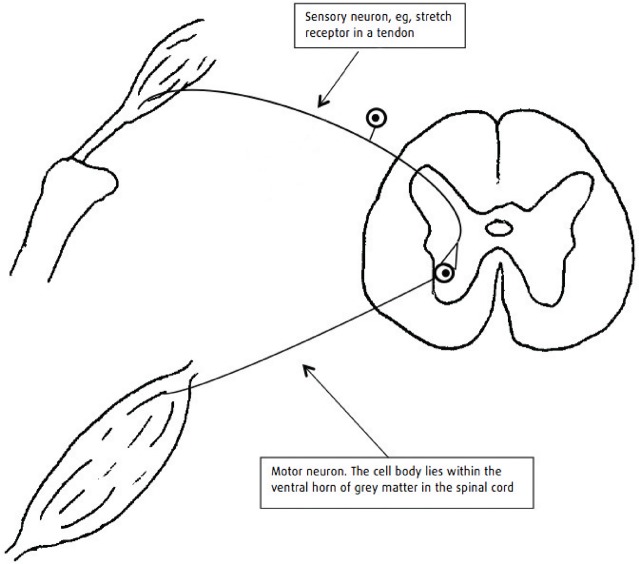
Simple reflex arc. The reflex arc provides the basis for neurological examination of the limbs and trunk. At its most simple, it involves just two neurons – the afferent (sensory) neuron and the efferent (motor) neuron. For example, increased tension in a tendon is detected by stretch receptors resulting in activation of the afferent neuron, which, through a direct synapse to a motor neuron, increases motor neuron activity and, thus, muscular contraction

**Fig 2:**
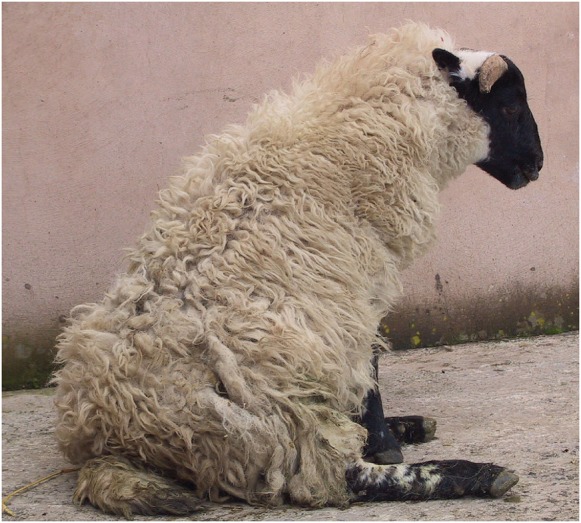
Scottish Blackface lamb with a compressive thoracolumbar lesion. Spinal lesions in the thoracolumbar region often result in animals adopting a dog-sitting position. The hindlimb myotatic reflexes (eg, patellar reflex) are usually exaggerated in such cases

**Fig 3:**
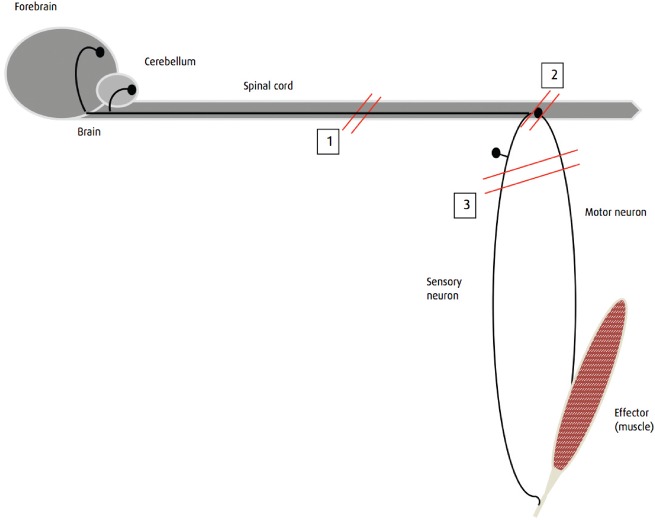
Schematic diagram showing upper motor neuron (UMN) and lower motor neuron (LMN) lesions relative to the spinal cord. UMN lesions (1) may occur anywhere cranial to the nerve roots that give rise to the nerves involved in the reflex/limb/muscle group in question. They disrupt the descending (inhibitory) axons that originate in the brain and also disrupt ascending pathways conveying sensation and proprioceptive information. LMN lesions may be central (2) or peripheral (3). Lesions in these locations may affect just motor neuron function or may affect both motor neurons and sensory neurons, leading to a loss of sensation as well as paresis/paralysis

Neural control of the limb musculature can be divided into local reflex arcs (Fig 1) and a descending modulation of these, which largely reduces the responsiveness of the reflex arc. Disruption of the efferent arm of a local reflex arc (a lower motor neuron [LMN] lesion) results in hyporeflexia, flaccid paralysis and rapid loss of muscle mass. Such lesions affect (from the peripheral to central aspects) the neuromuscular junction, the nerve trunk itself, the plexi where these nerves form from segmental spinal nerves, the ventral nerve roots of the segmental nerves or the spinal cord where the nerves originate. Disruption of the descending neurons (an upper motor neuron [UMN] lesion) removes inhibition from the reflex arc resulting in hyper-reflexia, spastic paralysis and a slower loss of muscle mass (Fig 2). Such a lesion occurs in the spinal cord cranial to the segment where the nerve roots give rise to the nerves involved in the reflex arc. It is accompanied by loss of conscious sensation and proprioception caudal to the lesion. An LMN lesion may have only motor deficits (damage to peripheral nerve trunks will result in sensory deficits distal to the lesion) and may occur in the spinal cord or involve only the peripheral nervous system; UMN lesions are always central (Fig 3). This distinction is important because the peripheral nervous system has a greater regenerative capacity than the central nervous system (CNS).

At the end of the neurological examination it should be possible to locate the lesion within one of the following functionally anatomical groups:

▪ Forebrain

▪ Cerebellum

▪ Vestibular system

▪ Brainstem

▪ C1-C5

▪ C6-T2

▪ T3-L3

▪ L4-S3

▪ Peripheral nerve/muscle/neuromuscular junction.

## Peripheral nerve lesions

Peripheral lesions may affect either a single nerve trunk or multiple nerves within the same limb. Generalised peripheral neuropathies do occur (botulism, for example) but are rare in sheep. The number of nerves affected and the degree of nerve disruption affect the clinical signs seen.

The large myelinated A fibres convey motor signals to the muscle fibres and also carry proprioceptive information and are the most vulnerable to injury (eg, hypoxia). C fibres are the smallest fibres and have the lowest conduction speed but are the more refractory to injury. They convey temperature, itch and deep pain sensation.

A mild insult may result in neuropraxia – a temporary dysfunction of the axon. Spontaneous recovery is relatively rapid, within a matter of days. If the insult is more severe, resulting in transection of the axons, then recovery is possible but this relies on the myelin sheath and connective tissue surrounding the nerve remaining intact. The axon can re-extend within this framework but this is a slow process. This type of injury is known as axonotmesis. If the nerve structure (ie, axons and myelin sheaths) is highly disrupted (neurotmesis), then this axonal regeneration may well not reach its intended target, leading to a permanent loss of function. Whether reinnervation occurs depends on the size of the gap between the proximal and distal nerve segments. Neuroma (a swelling resulting from haphazard regrowth of the axons and often a cause of discomfort) is one potential outcome of failed regeneration. Functional deficits may also arise due to ‘reorganisation’, in which suboptimal repair results in the axon terminating in an ‘off-target’ site.

Presenting signs and common causes of some peripheral nerve lesions are listed in Table 2.

**Table 2:** Presenting signs and potential causes of peripheral nerve lesions

Problem	Presenting signs	Potential causes
Brachial plexus avulsion	Dropped elbow, passively flexed fetlock and carpus, generalised decreased limb tone (unable to bear weight), easily abducted limb, decreased limb sensation, decreased extensor carpi radialis (ECR) reflex, possible disruption of the panniculus reflex, possible Horner's syndrome	Forelimb stuck in fence/gate, ill-fitting harness in rams
Radial nerve damage	Knuckling of the fetlock and carpus but the animal can bear weight, decreased sensation of antebrachium, metacarpus and foot, possible flexed elbow if the damage is higher up, decreased ECR reflex	Prolonged lateral recumbency, trauma to the lateral aspect of the forelimb
Sciatic nerve damage	Decreased sensation of the whole limb except the medial aspect, decreased tone except in the quadriceps and psoas musculature, decreased withdrawal reflex, intact or exaggerated patellar reflex	Iatrogenic damage due to injection into the hamstrings or caudal gluteal areas
Femoral nerve damage	Animal is unable to bear weight (if bilateral, the animal exhibits a frog-legged position), inability to extend the stifle, decreased patellar reflex, decreased skin sensation over the craniomedial crus	Hindlimb stuck over a gate/fence, trauma to the cranial aspect of the thigh, hyperextension of the hip
Distal limb nerve damage	Variable, depending on precisely which nerves are involved and the degree of damage	Trauma to a distal limb (eg, entrapped in wire)

Obturator nerve paralysis, which presents as weakness of the adductor and gracilis muscles leading to an inability to adduct the hindlimbs, is a known sequela to dystocia in cattle but is rare in sheep.

As spontaneous recovery is possible, and as long as any rectifiable problem has been attended to (eg, a foreign body removed, an abscess drained), then supportive care and minimising the accidental self-trauma that accompanies limb flaccidity can yield satisfactory results. For example, where fetlock ‘knuckling’ occurs, splinting of the distal limb to prevent trauma to the dorsum of the foot is indicated. Spontaneous recovery may take many weeks and clients must be prepared for a long wait without a guarantee of recovery.

## Spinal lesions

Spinal lesions have a poorer prognosis than peripheral nerve lesions due to the difference in response to injury. Unlike the nerve regeneration witnessed peripherally, axonal regeneration is poor in the central nervous system due to the physical barrier presented by the glial scar and the local factors produced by CNS glia and myelin debris that are non-permissive for growth (Kotter and others 2006).

The clinical signs associated with lesions of each of the major spinal cord segments are listed in Table 3 and will depend on the severity of disruption of the spinal cord and the precise location of the lesion. Reflexes and sensation may be reduced or completely absent and a degree of difference may occur between the left and right sides if the lesion is lateral to the spinal cord (eg, a perivertebral abscess extending into the spinal canal from the left may cause more severe deficits on the left side than the right side). Cervical lesions often produce more obvious gait deficits in the hindlimbs than the forelimbs.

**Table 3:** Clinical signs associated with lesions of the spinal cord

Lesion location	Forelimb intrinsic reflexes	Forelimb conscious sensation and proprioception	Hindlimb intrinsic reflexes	Hindlimb conscious sensation and proprioception	Notes
C1-C5	Increased	Decreased	Increased	Decreased	
C6-T2	Decreased	Decreased	Increased	Decreased	Disruption of the spinal cord at the level of T1-T3 could theoretically abolish the origin of the sympathetic supply to the head leading to Horner's syndrome
T3-L3	Normal	Normal	Increased	Decreased	Schiff–Sherrington phenomenon may occur with severe acute lesions and involves an increased thoracic limb extensor tone with retention of voluntary movements and normal proprioceptive placing in these limbs, accompanied by hindlimb paresis/paralysis
L4-S2	Normal	Normal	Decreased	Decreased	
S2 onwards	Normal	Normal	Normal	Normal	Cauda equina syndrome seen: decreased tail tone, altered perineal sensation, disrupted perineal reflex, passive bladder distension

Particular lesions are more commonly seen in certain spinal locations and further diagnostic evidence can be gathered from the history, signalment and diagnostic tests (see below). Common spinal lesions in sheep are listed in Table 4.

**Table 4:** Common causes of neurological signs that indicate a spinal cord lesion

Lesion	Location	Common signalment	Clinical signs	Treatment	Cause
Atlanto-occipital joint infection	Atlanto-occipital joint	Usually in young lambs (within the first two weeks of life)	Low head carriage, ataxia (hindlimb more noticeable), tetraparesis, neck pain (see Video 2)	Single injection of dexamethasone and seven days of procaine penicillin	Effectively a form of neonatal septic polyarthritis (joint ill), which is frequently caused by *Streptococcus dysgalactiae*
Compressive cervical myeloencephalopathy (wobbler syndrome)	Cervical	Texel and Beltex rams	Ataxia of all four limbs (see Video 3), paresis, may progress to tetraplegia	None	Fatty nodules on the floor of the spinal canal. There appears to be a hereditary component, so affected animals should not be bred from
Traumatic injury	Anywhere, frequently cervical	Often in rams that have been fighting	Clinical signs consistent with location, greater severity in cases of fracture	Mild cases frequently recover within a few days. Treatment should involve isolation, confinement and anti-inflammatory medication. Cases with unstable or displaced fractures should be euthanased	Often due to fighting among rams or other trauma (eg, being hit by car)
Vertebral body osteomyelitis	Frequently thoracolumbar but may be anywhere along the spinal cord	May be seen in any age of sheep but many cases involve lambs aged four to 12 weeks. It is usually a sporadic problem affecting individual animals	Dependant on location, onset usually sudden. If thoracolumbar, the animal will be bright, alert and responsive but with paresis or paralysis of the hindlimbs. It will often sit like a dog and move by pulling itself along using its forelimbs (see Videos 5 and 6)	Euthanasia	Thought to be due to bacterial infection of the vertebral body following bacteraemia but this is unproven
Delayed swayback	Clinical signs indicate a cranially progressive problem	Most commonly in lambs aged four to 12 weeks. It is usually a flock problem	Ascending paresis/paralysis and ataxia, gradual onset (see Video 7)	If mildly affected, an animal may make slaughter weight. If severely affected, the animal should be euthanased	Copper deficiency during pregnancy
Sarcocystosis	Clinical signs frequently consistent with a thoracolumbar lesion	Usually lambs about one year of age. Multiple animals are usually affected	Hindlimb paresis/paralysis, may be tetraparetic (see Video 8)	Mild cases may recover with nursing care but severe cases should be euthanased	Ingestion of sarcocyst oocysts (shed in dog faeces) by naïve animals

## Scrapie

Scrapie is a transmissible encephalopathy of sheep and goats. Paresis and ataxia, especially of the hindlimbs, are common clinical signs (Healy and others 2003, Jeffrey and Gonzalez 2007); quadriplegia and recumbency are seen at a later date. Other clinical signs include separation from the rest of the flock, depression, anxiety or hyperexcitability, head tremor, low head carriage, pruritus (including a ‘nibble’ response to stimulation of the back), weight loss, bruxism, cud-dropping and an absent menace response (Healy and others 2003, Konold and Phelan 2014). Most of the sheep that are affected are more than two years of age (Jeffrey and Gonzalez 2007).

Definitive diagnosis is by detection of the protease-resistant prion protein (PrPsc) in the brain on postmortem examination. Infection can be detected by the isolation of PrPsc from lymphoid tissue biopsies (eg, tonsillar tissue or rectal mucosa).

If scrapie is suspected in the UK, then the local APHA office must be notified.

## Maedi-visna

Maedi-visna is caused by maedi-visna virus (MVV), a small ruminant lentivirus. A variety of clinical syndromes follow infection, including progressive weight loss accompanied by dyspnoea and tachypnoea due to a lymphoid infiltrate of the interalveolar septa (maedi), indurative mastitis and, more rarely, arthritis and a neurological form (visna) (Pritchard and McConnell 2007).

**Fig 4:**
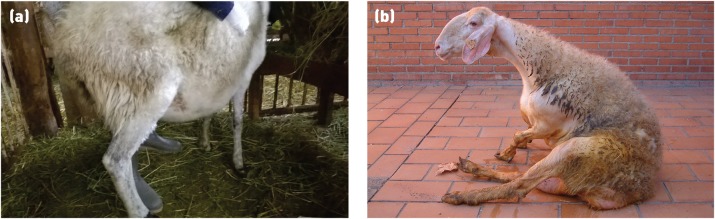
Limb paralysis due to infection with maedi-visna virus (MVV). Early in the disease course animals often show unilateral hindlimb paresis (a). The disease then progresses to ataxia, hindlimb paralysis (b), recumbency and death. Animals affected with visna are usually a small subsection of those infected with MVV in a flock (Picture b: Valentín Pérez and Julio Benavides)

Visna is most commonly seen in heavily infected flocks with widespread maedi, but there is some suggestion of different strains of MVV having different degrees of neurotropism (Payne and others 2004, Benavides and others 2006). Early clinical signs include unilateral paresis of a hindlimb (Fig 4) progressing to ataxia, hindlimb paralysis, recumbency and death. Other signs include fine head tremor, circling, progressive head tilt, visual impairment and separation from the rest of the flock (Pritchard and McConnell 2007). Affected animals are usually four to five years old but rapidly progressive disease has been reported in lambs as young as four months of age (Benavides and others 2007). Visna is more rapidly progressive than maedi, with affected animals dying or being euthanased within weeks of the onset of clinical signs. As visna is usually seen in flocks in which maedi is already present, suspicion may be high on clinical signs alone.

Affected animals are seropositive. Histopathology findings at postmortem examination include non-suppurative encephalitis affecting the cerebellum. MVV can be demonstrated immunohistopathologically or by PCR in the cerebellum at postmortem examination.

## Ancillary diagnostic techniques

Additional diagnostic techniques can help to confirm a diagnosis; however, those that are within the remit of specialist referral centres, such as computed tomography (CT) and magnetic resonance imaging (MRI), will not be considered here.

### Cerebrospinal fluid sampling

Cerebrospinal fluid bathes the CNS and protects it from mechanical damage. It is produced at the choroid plexi within the ventricles of the brain. Under normal circumstances it is a clear, colourless fluid with a very low cell count, consisting mainly of macrophages. It can be sampled from two sites – the cisterna magna, which lies deep to the foramen magnum of the skull, and the lumbosacral space. Sampling from the cisterna magna requires deep sedation or general anaesthesia and carries with it the risk of iatrogenic brain damage. Sampling from the lumbosacral space is much easier, can be performed on the conscious animal and carries a lower risk of iatrogenic damage. Box 3 describes the sampling method. As there is rarely a significant difference between the samples obtained from either site, lumbosacral sampling is preferred in the majority of cases.

Box 3: **Technique for sampling cerebrospinal fluid**

▪ Place the sheep in sternal recumbency with an assistant kneeling in front and grasping the animal's hocks. Positioning may be assisted by sedation of the animal

▪ Pull the hocks forward to extend the leg, flex the hip and flex the caudal spine; this widens the lumbosacral space. The landmarks that can be seen are the two wings of the ilium and the dorsal spinous processes

▪ With the thumb and ring finger on the wings of the ilium and the index finger lying along the dorsal spinous processes, feel the lumbosacral space as a depression just caudal to a line drawn between the two iliac crests, as shown in the photograph on the right

▪ Prepare this area surgically and place a small bleb of local anaesthetic beneath the skin

▪ Advance a needle slowly, perpendicular to the skin. A decrease in resistance will be felt as the point passes through the interarcuate ligament. The needle should be cautiously advanced until cerebrospinal fluid wells up in the hub

▪ Collect the fluid as it drips from the needle hub or attach a 5 ml syringe and apply gentle suction. No more than 2 ml should be aspirated

▪ Place the fluid in an EDTA tube immediately (Scott 1993)[Fig INPRACTH5547F9]

**Figure INPRACTH5547F9:**
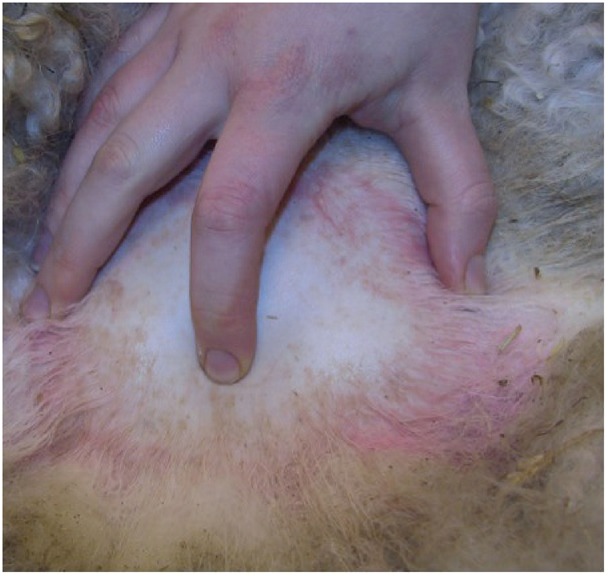
The lumbosacral space can be felt as a depression just caudal to a line drawn between the two iliac crests

Changes to the CSF that are relevant include:

▪ Blood staining – this is due to very recent haemorrhage or artefactual due to iatrogenic damage of vessels within the spinal canal. Sedimentation over several hours or centrifugation will clear the colour;

▪ Xanthochromic appearance – yellow/orange discolouration is indicative of past haemorrhage; centrifugation will not clear the colour. Haemosiderophages may be seen among the cells present;

▪ Frothy appearance, which forms a stable foam when shaken – this indicates that the protein content is increased;

▪ Turbidity – the cell count is much higher than normal (eg, in bacterial meningitis; in this case the fraction of neutrophils is also increased).

If there are no gross changes, the fluid should be submitted for laboratory analysis of the protein concentration because disruption of the flow of CSF (as occurs with a space-occupying lesion compressing the spinal cord) results in an increase in the protein concentration of the CSF caudal to the lesion – a phenomenon known as Froin's syndrome (Scott and Will 1991). This increase is too small to be detected using a refractometer.

Analysis of the cell types present within the CSF can also aid in diagnosis and can be performed at a low cost using sedimentation with standard Diff-Quik staining of the sediment and simple light microscopy (see Lowrie and Anderson [2011] for a full description of this technique). Sarcocystis merozoites have also been identified in CSF samples from sheep with neurological signs (Formisano and others 2013).

### Radiography

**Fig 5:**
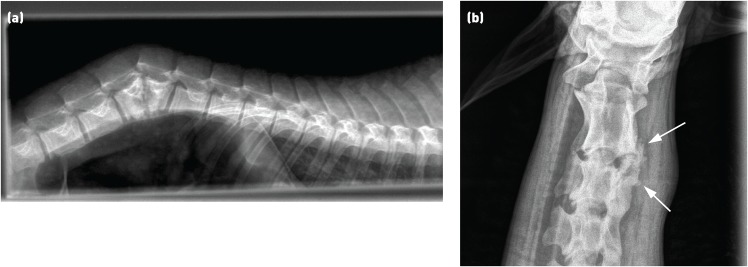
Radiograph showing (a) vertebral body destruction and disruption of the normal spinal architecture due to vertebral body osteomyelitis affecting the lumbar vertebrae. (b) New bone formation and loss of normal architecture (arrows) around the C2-C3 articulation on the right-hand side caused by a perivertebral abscess

Radiography of the spine can detect gross distortions of the bones of the vertebral column or the limbs. The clarity of images of sheep is often limited by the fleece, which adds artefacts and prevents placement of the plate as close to the area in question as is possible in other species. Nonetheless, it can be helpful in the detection of fractures or gross changes associated with osteomyelitis or perivertebral infections (Fig 5).

### Ultrasonography

Ultrasonography can be used to detect perivertebral abscesses in the cervical area, for example, those derived from intramuscular injection in the neck using dirty needles or irritant substances (see Video 4). It may also be used to assess the degree of urinary bladder distension (Scott and Sargison 2010) that may occur as a result of damage to the pelvic nerves (eg, in cauda equina syndrome).

### Myelography

**Fig 6:**
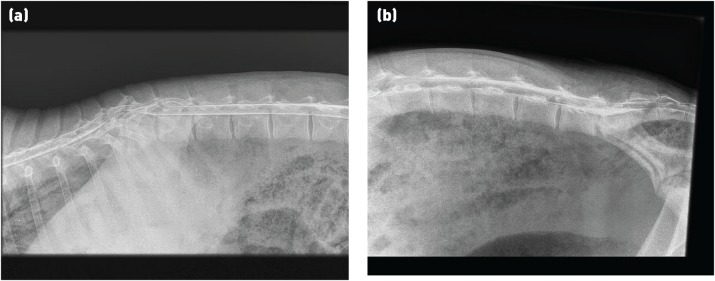
Myelograph showing (a) narrowing and dorsal deviation of both the dorsal and ventral columns of contrast material at the level of T11-T12. This lamb had vertebral body osteomyelitis resulting in erosion of vertebral bodies of T11 and T12. (b) Pooling of contrast material at the injection site (the lumbosacral junction). This myelograph has been taken too soon after administration of the contrast material as the dorsal contrast column is well established only as far as L3. The lesion in this case was at T10

The injection of contrast material into the epidural space can be performed at the same lumbosacral site as for CSF sampling (Fig 6). Approximately 5 ml injected into a two- to three-month-old lamb (15 to 20 kg) will allow imaging of the thoracolumbar spine. Elevating the hindquarters after injection aids cranial spread of the contrast material. The dorsal and or ventral columns on the lateral view will appear narrowed if the spinal cord is compressed. Dorsoventral projections are often very hard to interpret in sheep.

While this technique can aid the detection of spinal cord compression that is not accompanied by bony changes, it may not add any further information on localisation and is probably too costly for all but the most valuable animals.

## Flock implications

The diagnosis of certain causes of paresis or paralysis may have implications for the flock as a whole. The response may be governed by law, as with suspected scrapie, or will have to be worked out between the vet and the flock owner.

**Fig 7:**
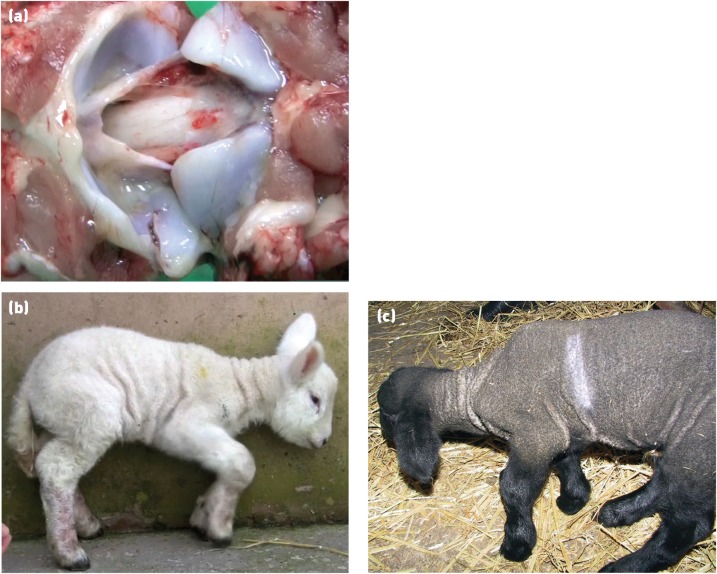
Atlanto-occipital joint infection. (a) Sepsis of the atlanto-occipital joint appears to be part of the same septic polyarthritis complex as the more frequently seen septic arthritides of limb joints (eg, carpus and tarsus). (b) and (c) Affected lambs show neck pain, low head carriage, ataxia and tetraparesis, which manifests frequently as flexion of the forelimbs

Some sporadic problems, such as neuropathies due to injury or occasional cases of vertebral body osteomyelitis, may not require any changes to flock management if they occur in singly; however, larger numbers of these problems should prompt careful investigation to identify the risk factors. For example, if many cases of atlanto-occipital joint infection (Fig 7) are seen in a flock, then colostrum intake, lambing shed hygiene and tick challenge, as well as the incidence of related problems such as infectious polyarthritis, should be evaluated.

Other problems, such as swayback (which rarely occurs singly and has a management-related cause [insufficient copper available in the diet of the pregnant ewe]), should automatically prompt discussion of the cause of the disease and control of risk factors pertaining to it.

Causes of paresis and paralysis with flock implications and examples of control measures are summarised in Table 5.

**Table 5:** Common causes of paresis and paralysis, and examples of flock control measures

Problem	Examples of control measures at flock level
Atlanto-occipital joint infection	Improve colostrum intake, navel dipping and lambing shed hygiene. Tick control may be appropriate if the involvement of tick-borne fever or tick pyaemia is suspected
Swayback	Increase dietary provision of copper for pregnant ewes (beware overcompensation and subsequent copper toxicity)
Vertebral body osteomyelitis	Aetiology not fully elucidated but any risk factors for bacteraemia or immunosuppression should be identified and controlled
Sarcocystosis	Identify the at-risk pasture/feedstuff and remove young animals from it. Prevent dog access to sheep carcases and ensure feedstuffs cannot become contaminated by dog faeces
Abnormal localisation of *Taenia multiceps* lesions	This is a rare presentation; classical gid is also likely to be seen. Control is as for sarcocystosis and includes regular worming of farm dogs with praziquantel
Compressive cervical myeloencephalopathy (‘wobbler syndrome’)	Do not breed from affected rams (and ewes), identify and outcross or remove at-risk family lines
Traumatic injury	Identify and remove the causes (eg, take care when mixing rams, ensure any trailers used to transport animals are fit for purpose)
Perivertebral abscessation	Identify the causes and, where abscesses are thought to result from iatrogenic injury, review the injection site, technique, equipment and product
Maedi-visna	Test the flock serologically to establish prevalence levels – test and cull policies have been successful in eliminating disease. Complete depopulation followed by repopulation from accredited disease-free sources is also possible. If neither option is practical, mitigate the spread and effects of the disease by ensuring a relatively young age profile to the flock, avoid or limit the period of housing and keep good records to identify and remove offspring or ewes identified as being clinical cases of maedi–visna
Brachial plexus damage	Identify and remove the inciting causes, for example, poorly fitted raddle harnesses on rams, poor injection site choice, poorly designed pens or fences that allow entrapment of limbs, and unsympathetic handling causing panicking sheep to become entrapped or trampled
Damage to individual nerve trunks	Control is as for brachial plexus damage

## Summary

A logical approach incorporating a basic neurological examination can allow localisation of lesions causing paresis or paralysis. This, alongside history and signalment, will often allow a presumptive diagnosis that is sufficient to provide a prognosis and decide on treatment. Further diagnostic tests will allow confirmation of diagnoses made on clinical grounds. The individual prognosis and flock implications of the various causes of limb paresis/paralysis in sheep are widely variable and an accurate diagnosis is vital in ensuring the welfare of individuals and the health of the flock.

Diagnosing limb paresis and paralysis in sheep

A number of videos accompany the online version of this article at: http://inpractice.bmj.com

**Video 1:** Ewe with paresis suffering from hypocalcaemia. After treatment, the tremors of the head are more pronounced when the ewe stands and the tremors of the rest of the body become apparent

**Video 2:** This two-week-old lamb displays tetraparesis, ataxia (especially of the hindlimbs) and restricted neck movements. The lesion was localised to the region C1-C5 and, given the age of the lamb, a presumptive diagnosis of atlanto-occipital joint infection was made. The lamb was treated with an injection of dexamethasone at presentation and seven consecutive days of procaine penicillin, and made a full recovery

**Video 3:** Two texel rams with compressive cervical myeloencephalopathy. Note the ataxic gait and the scuffing of the toes indicating paresis

**Video 4:** This gimmer presented with a two-week history of paresis progressing to tetraplegia. Note the intact panniculus reflex and the normal-to-exaggerated myotatic reflexes of the fore- and hindlimbs. Clonus is present following stimulation of the patellar reflex of the right hindlimb. The lesion was localised to region C1-C5 and careful palpation of the neck revealed a swelling on the right-hand side. Ultrasonography suggested a perivertebral abscess (a radiograph from this ewe can be seen in Fig 5)

**Video 5:** Lamb with thoracolumbar vertebral osteomyelitis, showing paresis of the hindlimbs. This lamb had a history of sudden-onset hindlimb dysfunction

**Video 6:** Lamb with thoracolumbar vertebral osteomyelitis. This lamb is completely paralytic in the hindlimbs. This lamb had a history of sudden-onset hindlimb dysfunction

**Video 7:** This lamb with delayed swayback shows hindlimb ataxia and paresis. The history was more slowly progressive than either lamb in Videos 5 and 6.

**Video 8:** This Scottish Blackface gimmer displays hindlimb paresis but has hindlimb myotatic reflexes of normal to increased intensity. The absence of a panniculus response over the lumbar flank suggested a lesion in the mid-thoracolumbar region of the spinal cord. Further diagnostic tests were unrewarding. No obvious lesions were detected at postmortem examination. A provisional diagnosis of thoracolumbar Sarcocystis myeloencephalitis was made. Frustratingly, gross lesions are not seen in cases of sarcocystosis at postmortem examination
